# Incidence of Occult Lymph Node Metastasis in Primary Larynx Squamous Cell Carcinoma, by Subsite, T Classification and Neck Level: A Systematic Review

**DOI:** 10.3390/cancers12041059

**Published:** 2020-04-24

**Authors:** Alvaro Sanabria, Jatin P. Shah, Jesus E. Medina, Kerry D. Olsen, K. Thomas Robbins, Carl E. Silver, Juan P. Rodrigo, Carlos Suárez, Andrés Coca-Pelaz, Ashok R. Shaha, Antti A. Mäkitie, Alessandra Rinaldo, Remco de Bree, Primož Strojan, Marc Hamoir, Robert P. Takes, Elisabeth V. Sjögren, Trinitia Cannon, Luiz P. Kowalski, Alfio Ferlito

**Affiliations:** 1 Department of Surgery, School of Medicine, Universidad de Antioquia/Hospital Universitario San Vicente Fundación, Medellín 050010, Colombia; alvarosanabria@gmail.com; 2 CEXCA Centro de Excelencia en Enfermedades de Cabeza y Cuello, Medellín 050021, Colombia; 3 Department of Surgery, Memorial Sloan Kettering Cancer Center, New York, NY 10065, USA; shahj@mskcc.org (J.P.S.); shahaa@mskcc.org (A.R.S.); 4 Department of Otorhinolaryngology, The University of Oklahoma Health Sciences Center, Oklahoma City, OK 73117, USA; Jesus-Medina@ouhsc.edu; 5 Department of Otorhinolaryngology, Mayo Clinic, Rochester, MN 55902, USA; olsen.kerry@mayo.edu; 6 Department of Otolaryngology-Head and Neck Surgery, Southern Illinois University School of Medicine, Springfield, IL 32952, USA; kthomasrobbins@gmail.com; 7 Department of Surgery, University of Arizona College of Medicine, Phoenix, AZ 85259, USA; csilver@cox.net; 8 Department of Otolaryngology, Hospital Universitario Central de Asturias-ISPA, 33011 Oviedo, Spain; jprodrigo@uniovi.es (J.P.R.); acocapelaz@yahoo.es (A.C.-P.); 9 University of Oviedo-IUOPA, 33011 Oviedo, Spain; 10 Head and Neck Cancer Unit, CIBERONC, 28029 Madrid, Spain; 11 Instituto de Investigación Sanitaria del Principado de Asturias, 33011 Oviedo, Spain; csuareznieto@gmail.com; 12 Department of Otorhinolaryngology—Head and Neck Surgery, University of Helsinki and Helsinki University Hospital, FI-00029 HUS Helsinki, Finland; Antti.Makitie@hus.fi; 13 University of Udine School of Medicine, 33100 Udine, Italy; alessandra.rinaldo@uniud.it; 14 Department of Head and Neck Surgical Oncology, University Medical Center Utrecht, 3584CX Utrecht, The Netherlands; R.deBree@umcutrecht.nl; 15 Department of Radiation Oncology, Institute of Oncology, SI-1000 Ljubljana, Slovenia; pstrojan@onko-i.si; 16 Department of Head and Neck Surgery, UC Louvain, St Luc University Hospital and King Albert II Cancer Institute, 1200 Brussels, Belgium; marc.hamoir@uclouvain.be; 17 Department of Otolaryngology-Head and Neck Surgery, Radboud University Medical Center, 6500HB Nijmegen, The Netherlands; Robert.Takes@radboudumc.nl; 18 Department of Otolaryngology—Head and Neck Surgery, Leiden University Medical Centre, 2300 RC Leiden, The Netherlands; evsjogren@lumc.nl; 19 Department of Head and Neck Surgery and Communication Sciences, Duke University Health System, Durham, NC 27609, USA; Trinitia.Cannon@duke.edu; 20 Department of Otorhinolaryngology-Head and Neck Surgery, A.C. Camargo Cancer Center, 01509-900 São Paulo, Brazil; lp_kowalski@uol.com.br; 21 Department of Head and Neck Surgery, University of São Paulo Medical School, 05402-000 São Paulo, Brazil; 22 International Head and Neck Scientific Group, 35100 Padua, Italy

**Keywords:** larynx neoplasm, neck dissection, systematic review, glottis, supraglottis

## Abstract

Background: Larynx cancer is a common site for tumors of the upper aerodigestive tract. In cases with a clinically negative neck, the indications for an elective neck treatment are still debated. The objective is to define the prevalence of occult metastasis based on the subsite of the primary tumor, T classification and neck node levels involved. Methods: All studies included provided the rate of occult metastases in cN0 larynx squamous cell carcinoma patients. The main outcome was the incidence of occult metastasis. The pooled incidence was calculated with random effects analysis. Results: 36 studies with 3803 patients fulfilled the criteria. The incidence of lymph node metastases for supraglottic and glottic tumors was 19.9% (95% CI 16.4–23.4) and 8.0% (95% CI 2.7–13.3), respectively. The incidence of occult metastasis for level I, level IV and level V was 2.4% (95% CI 0–6.1%), 2.0% (95% CI 0.9–3.1) and 0.4% (95% CI 0–1.0%), respectively. For all tumors, the incidence for sublevel IIB was 0.5% (95% CI 0–1.3). Conclusions: The incidence of occult lymph node metastasis is higher in supraglottic and T3–4 tumors. Level I and V and sublevel IIB should not be routinely included in the elective neck treatment of cN0 laryngeal cancer and, in addition, level IV should not be routinely included in cases of supraglottic tumors.

## 1. Introduction

The larynx is the site for the second most common cancer of the upper aerodigestive tract. While in some countries the incidence of larynx cancer has risen in the last forty years, due to smoking and drinking, in the USA and Northern Europe it has decreased dramatically [1,2].

Adequate treatment of larynx cancer centers around local control of the primary tumor, as well as control of clinical or occult neck lymph node metastasis. If the patient presents with positive lymph nodes in the lateral compartment of the neck and the planned treatment option for the primary tumor is surgery, there is no doubt that the preferred treatment is a neck dissection most likely followed by radiotherapy alone or in combination with chemotherapy [3]. The lymph node levels to be included in the dissection and the radiotherapy fields depend on the level of positive nodes found by palpation and radiological examination, as well as the laryngeal subsite(s) involved by cancer and T classification of the tumor. However, there still is an ongoing discussion about which neck levels should be included [4,5,6,7]. On the other hand, in cases with a clinically negative neck, the indications for and the extent of an elective neck dissection or elective neck irradiation are still debated vigorously in the literature, the scientific community and multidisciplinary tumor board meetings. Although, depending mainly on the subsite and T classification of the primary tumor, the final decision making should take into account the expected survival advantages as a result of elective treatment of the neck, as well as the functional and esthetic morbidity that may result from it.

The rationale to offer elective treatment of the neck in patients with larynx cancer is the potential presence of occult lymph node metastases, which can result in recurrence after successful initial treatment of the primary tumor. However, it has been reported that the results with elective neck dissection or irradiation are not superior to those of “watchful” surveillance without neck surgery or radiotherapy in early stage laryngeal cancer [8]. It is also known that the social and psychological make-up of some patients with larynx cancer is such that when they present with a recurrence in the neck after a “watchful” waiting policy, it is often at an advanced stage and salvage treatment may not be successful. Consequently, many clinicians prefer performing an elective neck dissection/irradiation in spite of the fact that tumor recurrence owing to adverse biological characteristics of the cancer may develop even after a neck dissection and radiation therapy. Therefore, the procedure may not offer any advantage in survival and the morbidity associated with an elective neck dissection may decrease the quality of life [9,10]. 

Central to the discussion of elective treatment of the neck is the ability to predict correctly the likelihood of occult metastasis in a given patient and the neck node levels that are at highest risk of containing metastases, which, therefore, need to be removed or irradiated electively. Unfortunately, neither modern imaging techniques nor molecular marker analysis of the primary tumor, nor sentinel lymph node biopsy [5,11], are generally felt to be accurate enough to be considered the standard of care.

The objective of this review is to assess the available literature in order to define the prevalence of occult metastasis in larynx cancer based on three key factors: subsite of the primary tumor (glottic or supraglottic), T classification and neck node levels involved. Information about bilateral metastasis was not included. The data are intended to provide a rationale for the indications and extent of elective neck treatment based on the specific factors analyzed.

## 2. Results

The primary search found 2025 studies. Only 36 studies with 3803 patients fulfilled the inclusion criteria for the review and are presented in Table 1 [10,12,13,14,15,16,17,18,19,20,21,22,23,24,25,26,27,28,29,30,31,32,33,34,35,36,37,38,39,40,41,42,43,44,45,46]. Most exclusions were based on indeterminate outcomes or because the primary treatment was with radiotherapy/chemoradiotherapy. Ten studies were excluded because cN+ patients were also included in the sample [47,48,49,50,51,52,53,54,55,56] and one because it was not possible to get full text [57]. Data from Deganello et al. [10], Pinilla et al. [23] and Zhang et al. [31] reported individual data about subsites and were divided in order to include their cases in different categories [glottic, supraglottic or larynx, not otherwise specified (LNOS)] (Table 1). Three studies from the same center had overlapping patients but they were analyzed independently because the numbers, outcomes and objectives were different [32,33,35]. 

All the studies were case series, except the case control study by Djordjevic et al. [45]. Eight studies were prospective [20,25,27,29,36,39,42,45]; fifteen studies analyzed the larynx without specification of occult metastasis by subsite [10,13,15,20,24,25,26,27,29,34,37,39,40,43,46]; nineteen analyzed supraglottic tumors [10,12,14,16,18,19,22,23,28,30,31,32,33,35,36,38,42,44,45]; and six, glottic tumors [10,17,21,23,31,41]. Katilmis et al. included a small number of hypopharynx tumors (13/213) [34].

### 2.1. Description of Patients Included in the Studies

In total, 2226 patients had supraglottic tumors, 412 patients had transglottic tumors and 776 had glottic tumors with or without extension to other sites, such as the subglottis or supraglottis. Two studies (389 patients) did not report distribution by subsite [24,26]. In total, 1643 patients were classified as T1–2 and 1785 as T3–4. In two studies (366 patients) it was impossible to obtain data on clinical stage [24,39]. Regarding treatment, 852 patients underwent partial laryngectomy while 675 had total laryngectomy. Thirteen studies did not report the surgical procedure done for the primary tumor [10,24,25,27,28,31,32,33,37,39,40,43,45]. 

### 2.2. Methodological Quality

**Figure 1:**
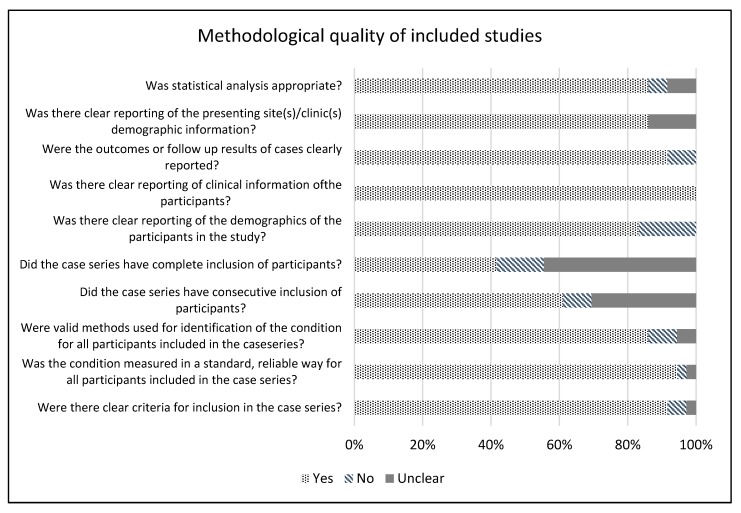
Methodological quality of the included studies.

The methodological quality of the included studies was moderate to good. The mean score was 8.3 ± 1.4 over a maximum score of 10. Only 4/36 (11%) of the studies scored lower than 7. The item with the lowest score was “Did the case series have complete inclusion of participants?”, where 21/36 (58%) studies had a no/unclear result, followed by “Did the case series have consecutive inclusion of participants?”, where 14/36 (39%) had a no/unclear result (Figure 1). 

### 2.3. Rate of Occult Metastasis

All studies, except two [34,39], reported an overall rate of occult metastasis of 18.7% (711/3803 patients). The pooled incidence was not calculated due to the expected clinical and statistical heterogeneity by subsites.

#### 2.3.1. Supraglottic Tumors

Nineteen studies specified supraglottic tumors [10,12,14,16,18,19,22,23,28,30,31,32,33,35,36,38,42,44,45]. The calculated pooled incidence of LN metastases was 19.9% (95% CI 16.4–23.4, I2 = 65%) (Figure S1). More than half (56.1%) of the patients were classified as having a T1–2 tumor. The rate of occult metastases varied widely among the reported series. Lawson et al. [36] showed a high risk of occult metastasis of 48.2%. Tu et al. [19], Ramadan and Allen [14] and Levendag and Vikram [12] showed rates of occult metastasis lower than 12%. No clear explanation for statistical heterogeneity was found. A subgroup analysis with studies that included >75% of T1–2 tumors [12,18,30,35,36,38] was done and the pooled incidence was 18.4% (95% CI 11.8–25.0, I2 = 54%) (Figure S2). 

#### 2.3.2. Glottic Tumors

**Figure 2:**
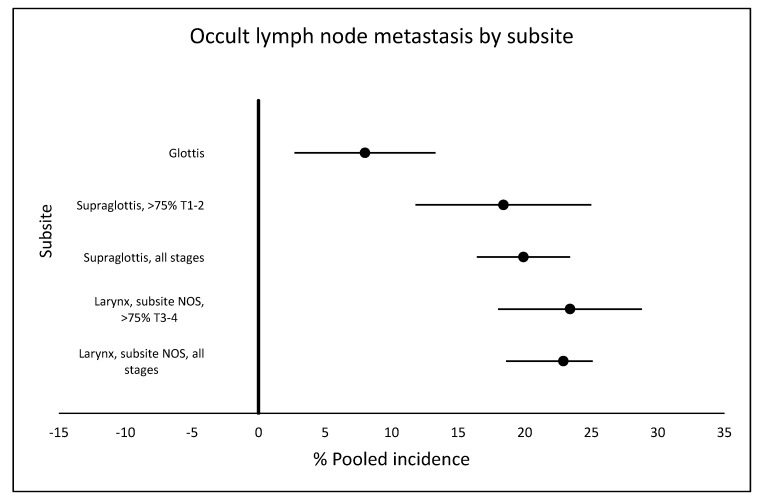
Comparison of pooled incidences of occult lymph node metastasis by subsite in cN0 larynx cancer. NOS: not otherwise specified

From the six studies that specified glottic tumors [10,17,21,23,31,41] approximately two thirds (65.6%) were classified as T1–2. They reported a pooled incidence for occult metastasis of 8.0% (95% CI 2.7–13.3, I2 = 81%) (Figure S1). Among these studies, Erdag et al. [41] reported an incidence for occult metastases of 0%, thus explaining the high statistical heterogeneity in this analysis. Figure 2 shows the comparison of occult lymph node metastasis by subsite. 

#### 2.3.3. Larynx Tumors without Specification of Subsite

Fifteen studies assessed larynx tumors without specifying the outcomes by subsite (NOS) [10,13,15,20,24,25,26,27,29,34,37,39,40,43,46]. Patients had tumors classified as supraglottic in 26.1%, transglottic in 24.8% and glottic/other site in 21.0% of the cases. One fifth (19.6%) were classified as T1–2 and more than half (54.0%) as T3–4. There was no information on subsite in 28.1% or stage in 26.4% of patients. These studies reported a pooled incidence of occult metastasis of 22.9% (95% CI 18.6–25.1, I2 = 58%) (Figure S1). A subgroup analysis of studies that included >75% of T3–4 tumors [15,25,26,37,40,43,46] was done, and the pooled incidence was 23.4% (95% CI 18.0–28.8, I2 = 44%) (Figure S3). 

### 2.4. Occult Metastasis by Lymph Node Level

**Figure 3:**
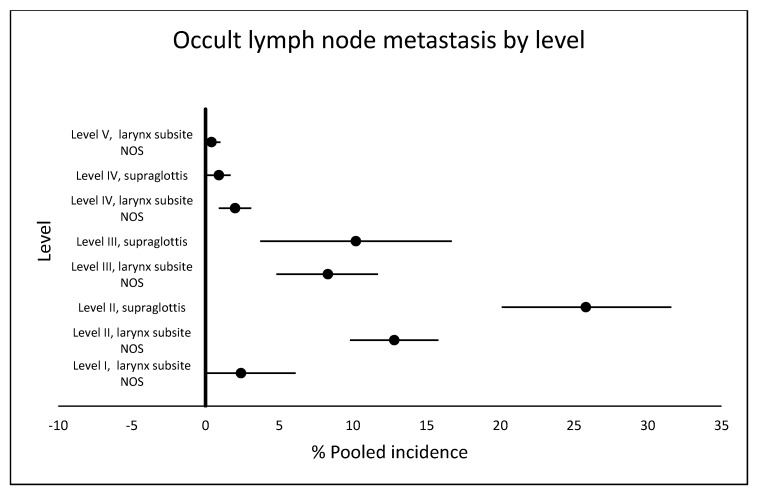
Comparison of pooled incidences of occult lymph node metastasis by level in cN0 larynx cancer. NOS: not otherwise specified

The presence of occult lymph node metastasis at level I was reported in only two studies [10,13], but in both there was no information about involvement of sublevels IA or IB. Level II involvement was reported in eleven studies [10,13,24,27,29,32,33,34,44,45,46], level III in eleven studies [10,13,24,27,29,32,33,34,44,45,46], level IV in seventeen studies [10,13,20,24,25,26,27,29,32,33,34,37,39,43,44,45,46] and level V in five studies [10,13,20,24,34]. Figures S4, S5 and Table S1 shows data about the studies and pooled incidence and Figure 3 shows the comparison of occult lymph node metastasis by level.

The studies of Candela et al. [13] and Djordjevic et al. [45] explain the statistical heterogeneity for the level III analysis and the study of Spriano et al. [24] explains the statistical heterogeneity for the level V analysis. 

### 2.5. Occult Metastasis by T Classification 

Five studies reported data on the T1 stage [16,21,23,29,31]. Five of 105 patients (4.8%) had occult metastasis. It was impossible to calculate a pooled incidence as Elo et al. [21], Pinilla et al. [23] and Lim et al. [29] did not observe any event in this category. 

Twelve studies reported data on the T2 stage [10,14,16,21,23,28,29,31,32,33,42,44]. For the subgroup with glottic tumors [21,23,31], the pooled incidence was 4.7% (95% CI 0.5–8.8, I2 = 13%). In the subgroup of supraglottic tumors [10,14,16,23,28,31,32,33,42,44], the pooled incidence was 16.5% (95% CI 14.8–18.3, I2 = 0%) (Figures S6 and S7). 

Fourteen studies reported data on the T3 stage [10,14,15,16,17,21,23,28,29,31,32,33,42,44]. For the subgroup of glottic tumors [10,17,21,23,31], the pooled incidence was 14.4% (95% CI 6.9–21.8, I2 = 11%). In the subgroup of supraglottic tumors [10,14,16,23,28,31,32,33,42,44], the pooled incidence was 23.8% (95% CI 18.6–28.9, I2 = 0%). For larynx subsite NOS, two studies [15,29] reported a pooled incidence of 16.5% (95% CI 0–38.6) (Figures S6 and S7)

**Figure 4:**
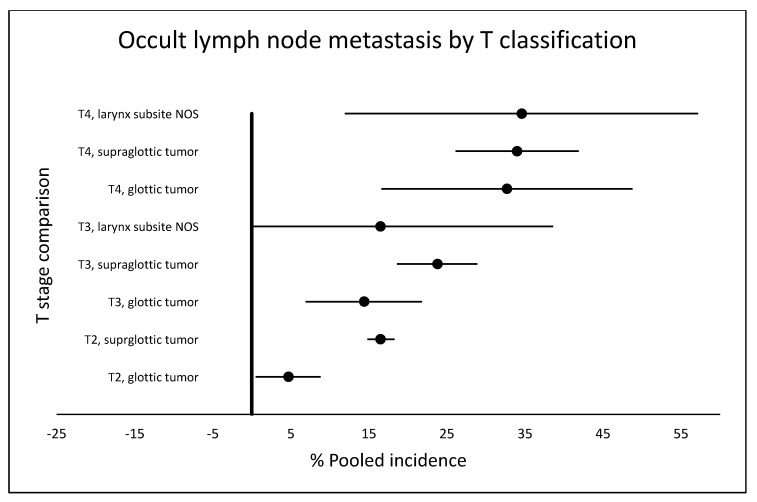
Comparison of pooled incidences of occult lymph node metastasis by T classification in cN0 larynx cancer. NOS: not otherwise specified.

Fourteen studies reported data on the T4 stage [10,14,15,16,17,21,23,28,29,31,32,33,42,44]. For the subgroup of glottic tumors [10,17,21,23,31], the pooled incidence was 32.7% (95% CI 16.6–48.8, I2 = 0). In the subgroup of supraglottic tumors, the pooled incidence was 34.0% (95% CI 26.1–41.9, I2 = 0). In the larynx subsite NOS, two studies [15,29] reported a pooled incidence of 34.6% (95% CI 11.9–57.2) (Figures S6 and S7). Figure 4 shows the comparison of occult lymph node metastasis by T classification.

## 3. Discussion

The role of elective neck treatment for larynx cancer patients with cN0 disease has been a subject of debate for many years [3,6,7]. This has been further complicated by the advent and effectiveness of non-surgical organ preservation protocols, as well as the introduction of endoscopic surgery as primary tumor treatment [5]. Moreover, since elective neck dissection may be a risk factor for pharyngocutaneous fistula after total laryngectomy, unnecessary neck dissection should be avoided [58]. The same holds true when radiotherapy is chosen as treatment, where extending the radiation fields unnecessarily can lead to serious complications that can adversely affect the quality of the patient’s life [59]. Given these issues, the present study was undertaken in an attempt to provide more homogeneous data from the available literature by quantitating the incidence of occult nodal disease based on mucosal site and T classification. Furthermore, an attempt was made to subdivide the presence of nodal disease based on neck level in order to elucidate the application of the various types of selective neck dissections. 

Lymphatic drainage patterns have been widely studied since the 1960s. The studies of Welsh et al. [60,61,62] demonstrated the differential drainage patterns of the larynx according to subsites. These studies demonstrate that the supraglottis is rich in lymphatic networks while for instance vocal cords do not have lymphatic vessels in their free margin. Liu et al. [63] demonstrated that the inferior surface of the vocal cord does have an important lymphatic network, with a similar drainage as the subglottis. These anatomical factors explain the pattern of distribution of lymph node metastasis, with levels II and III [6,20,39] being the most frequently involved. Metastasis to level VI is not usually reported, except in specific studies on primary subglottic tumors or advanced tumors involving this area.

In this review, the evaluation of occult lymph node metastasis by subsite demonstrated a higher risk for supraglottic (19%) compared to glottic tumors (8%). However, there exists a wide variation in the reported results. Unfortunately, the most common clinical and histopathological characteristics of the primary tumor provided by the studies analyzed do not help to elucidate the cause of such heterogeneity. For example, for larynx tumors NOS, Deganello et al. [10] reported a low incidence of occult metastasis of 12% while Tsushima et al. [46], Xu et al. [40] and Candela et al. [13] reported incidences higher than 32%. Heterogeneity was also found in supraglottic tumors. For example, Lawson et al. [36] found a 32.5% incidence of occult metastasis while Tu et al. [19] found 8.3%, but we are unable to explain these dramatic differences with the available data. Factors related to the indication for neck dissection, the policy of wait and see without providing the selection criteria used and the number of patients observed may explain these differences. Other factors responsible for this heterogeneity may be differences in diagnostic work-up and in the depth of histopathological examination of the neck dissection specimen. Routine histopathological examination of the neck dissection specimen can miss micrometastases in up to 15.2% and therefore the real incidence of occult metastases may be underestimated [64]. Serial sectioning and immunohistochemistry, as used in the examination of a sentinel lymph node biopsy, will detect micrometastases better, but is costly and can introduce a verification bias due to the higher number of sections. Finally, the use of more accurate diagnostic imaging techniques will result in lower reported incidence rates of occult metastases than the use of palpation only.

Analysis by T classification helps to reduce the variability among the patients studied and provides an improved understanding of the risk of occult nodal disease for each subsite. The assessment of occult lymph node metastases according to T classification confirms the increasing risk with increasing T stage. In fact, at the same T classification, supraglottic tumors always had a higher incidence in comparison to glottic tumors (16.5% vs. 4.7% in T2 tumors and 23.8% vs. 14.4% in T3 tumors). We found that early supraglottic tumors have a lower rate of occult metastasis than those with more advanced tumors. However, these differences were not significantly different. A remarkable result was found regarding glottic T3 tumors, which had a lower incidence than - T2 supraglottic tumors. 

The data from this analysis support the notion that neck levels I and V are at low risk for occult nodal disease in patients with cN0 laryngeal cancer. For level I, the incidence derived from the pooled data from two studies is 2.4%. However, it should be pointed out, as Candela et al. [13] state in their paper, that Level I is rarely involved and that the involvement they observe occurred usually in cases with neck node metastases in levels II, III or IV (75% of the time). It should also be noted that Level I involvement in their study occurred in T3 or T4 primary tumors exhibiting histologic extra laryngeal spread. While these two studies did not provide information about sublevel (IA or IB) involvement, others suggest that the risk of involvement of sublevel IA in cN0 larynx tumors is insignificant [4]. Similarly, the rate of occult metastases in level V was only 0.4%. Thus, routine elective treatment of these levels is not indicated in patients undergoing elective lymphadenectomy to remove potential occult disease among patients with laryngeal cancer and a cN0 neck. This recommendation is also supported by the results of a prospective trial that compared selective neck dissection of levels II to IV vs. modified radical neck dissection [65]. With regard to the inclusion of Level IV in elective treatment, our study found a very low rate of involvement of the nodes in this level in patients with supraglottic cancer, thus supporting the practice of not including them routinely in those cases. Similarly, our results regarding sublevel IIB indicated a 0.5% rate of occult disease supporting the practice of not including this sublevel routinely when performing a neck dissection for occult disease [66,67]. Some of these studies follow a uniform pattern regarding the lymphatic levels resected, while others use intraoperative findings or even frozen section results to guide the extent of the dissection. There is also still a lack of standardization in the nomenclature and in the execution of neck dissections, despite several initiatives to standardize them [68]. 

Even though there is a large number of publications dealing with the surgical treatment of the neck in larynx cancer, the quality of many studies is moderate, as shown in Figure 1. This is due, in part, to a selection bias of surgical studies, which may lead to a selection of more favorable primary tumors for surgery and those unfavorable for chemo/radiotherapy. Another factor is that most of these studies are retrospective case series, which are again prone to selection bias that can affect the pooled results. Other weaknesses are related to patient selection in individual studies. Furthermore, one study included a small number of patients with hypopharynx tumors, but the corresponding effect on the calculation of the pooled incidence is minimal [34]. Finally, during the more than 50-year period covered by our analysis, pre-therapeutic diagnostic procedures and protocols have changed dramatically, and thus the accuracy of the assessment of the neck stage prior to treatment. 

Obviously, the few factors analyzed in this review (T classification and laryngeal subsite) are clearly insufficient to accurately predict the risk of lymph node metastasis. Other tumor features also have been reported to be relevant in the development of lymph node metastases, such as the infiltrating pattern of growth of the tumor, “tumor budding” and lymphovascular and perineural invasion [69,70,71,72]; molecular changes, such as loss of N33, STK11 and TP53 [73]; as well as immune alteration, such as HLA-E overexpression [74]. However, the means to assess these characteristics are not readily available in all institutions. Furthermore, most of these can only be evaluated after resection of the primary tumor, which precludes its clinical use to make decisions about elective neck dissection. The use of an SLN biopsy could be explored in some of these cases [5,36]. 

## 4. Materials and Methods 

A search was conducted using the MESH and free-text terms “cancer”, “larynx” and “neck dissection” in the PubMed, Embase and LILACS databases for studies published between 1966 and March 2019. An expanded search was conducted, and references were explored to identify additional articles. We did not restrict the publication language. All selected studies provided the rate of occult metastases in cN0 larynx squamous cell carcinoma patients primarily treated. All abstracts were reviewed by the authors. Those related with the subject were selected for further analysis. Those studies in which it was not possible to isolate specific data about larynx tumors were excluded. A differential analysis was made when studies did not report outcomes by subsites. The main outcome measure was the incidence of occult metastasis in neck dissection specimens based on the mucosal subsite, T classification and neck levels involved. Studies dealing with organ-preserving approaches were not included.

Data were collected based on sample size, patient characteristics and outcomes. Data from each study were extracted and recorded in an Excel spreadsheet. The unit of analysis was the study and statistical analysis was performed with Excel (Microsoft). The pooled incidence (95% confidence interval (CI)) was calculated for each outcome with random effects analysis because this method is a conservative summary estimate and incorporates between- and within-study variance [75]. For data with 0 events, a correction with 0.1 was made in order to obtain calculations. Studies that reported the frequency of occult metastases but did not discriminate it by subsite were classified as larynx tumors not otherwise specified (LNOS). The others were classified as having glottic and supraglottic tumors. The rate of positive nodes by neck level was calculated from the number of positive lymph nodes in each level divided by the total number of patients. Statistical heterogeneity was calculated with the Higgins I2 statistic. This represents the amount of variation in incidence of the included studies and when found it is recommended to look for an explanation based on the clinical factors, methods or analysis. Results of the intervention effects are presented with a forest plot graph.

Methodological quality of the included studies was evaluated with the Checklist for Case Series of the Joanna Briggs Institute Critical Appraisal tools for use in systematic reviews [76].

## 5. Conclusions

In summary, the incidence of occult lymph node metastasis in cN0 larynx cancer depends on the subsite and T classification, where supraglottic and T3–4 tumors have the higher percentages. The final recommendations for the patient with larynx cancer and a clinically negative neck are the following: For cN0 T1/2 glottic tumors, neck dissection is not recommended; for cN0 T1/2 supraglottic tumors, level I, level IV, level V and sublevel IIB should not be routinely included in the elective neck treatment; and for cN0 T3/4 supraglottic tumors and cN0 T3/4 glottic tumors, level I, level V and sublevel IIB should not be routinely included in the elective neck treatment. This study did not allow us to make a recommendation about bilateral neck dissection in cN0 T1/2 supraglottic tumors. 

## Figures and Tables

**Table 1:** Characteristics of the studies included in the systematic review.

Author	Year	Country	Time Period	Study Type	Design	*N*	Subsite	cN+	T1–2/T3–4	Partial/Total Laryngectomy	% Occult Metastasis	Neck Level						T Classification							
												I	II	III	IV	V	IIB	T1	T1t	T2	T2t	T3	T3t	T4	T4t
Levendag and Vikram [12]	1987	USA	1965–1979	CS	R	79	S		79/0	31/48	10														
Candela et al. [13]	1990	USA	1965–1986	CS	R	78	LNOS		36/42	NR	29	4	15	16	7	2									
Ramadan and Allen [14]	1993	USA	1975–1986	CS	R	63	S		16/49	NR	7									1	4	3	8	3	9
Kligerman et al. [15]	1995	Brazil	1981–1989	CS	R	76	LNOS		0/76	1/75	23											16	56	7	20
Petrovic et al. [16]	1997	Yugoslavia	1976–1990	CS	R	161	S		90/71	NR	29							4	22	9	68	6	45	10	26
Yang et al. [17]	1998	USA	1984–1994	CS	R	92	G		71/21	NR	4							0	0	0	0	3	14	1	7
Güney and Yigitbasi [18]	1999	Turkey	1991–1996	CS	R	39	S		39/0	NR	9														
Tu [19]	1999	China	1976–1990	CS	R	155	S		51/91	128/34	13														
León et al. [20]	2001	Spain	1991–1997	CS	P	79	LNOS		23/56	NR	23				2	0									
Elo et al. [21]	2002	Hungary	1989–1999	CS	R	206	G		133/73	133/73	24							0	61	6	72	9	54	9	19
Amoros et al. [22]	2003	Spain	1977–1999	CS	R	164	S		103/61	NR	40														
Pinilla et al. [23]	2003	Spain	1983–1993	CS	R R	124	S		56/68	NR	34							0	6	9	50	9	42	16	26
						66	G		44/22	NR	9							0	1	1	43	6	18	2	4
Spriano et al. [24]	2003	Italy	1980–1997	CS	R	346	LNOS		NR	NR	59		56	33	5	10									
Coskun et al. [25]	2004	Turkey	1999–2002	CS	P	71	LNOS		12/59	NR	14				0		0								
Khafif et al. [26]	2004	Israel USA		CS	R	43	LNOS		0/43	0/100	9				1										
Elsheikh et al. [27]	2006	Egypt	2001–2004	CS	P	31	LNOS		19/12	NR	6		2	3	1		0								
Fiorella et al. [28]	2006	Italy		CS	R	106	S		50/56	NR	29									0, 16		19	53	2	3
Lim et al. [29]	2006	Korea	1997–2002	CS	P	73	LNOS		33/40	16/53	21		12	9	5			0	3	1	30	2	34	2	6
Rodrigo et al. [30]	2006	Spain	1975–1998	CS	R	108	S		108/0	108/0	16														
Zhang et al. [31]	2006	China	1997–2002	CS	R	72	S		36/36	NR	15							1	12	6	24	7	30	1	6
						38	G		13/25	NR	7									1	13	2	14	4	11
Cağli et al. [33]	2007	Turkey	1998–2006	CS	R	72	S		12/60	NR	16		16	7	1					1	12	10	44	5	16
Cağli et al. [32]	2007	Turkey		CS	R	58	S		22/36	NR	14		11	7	1					3	22	7	28	4	8
Katilmis et al. [34]	2007	Turkey	1998–2003	CS	R	224	LNOS		79/145	60/164			24	12	7	0									
Yüce et al. [35]	2009	Turkey	1991–2005	CC	R	71	S		71/0	67/4	9														
Lawson et al. [36]	2010	Belgium	2001–2004	CS	P	29	S		25/6	29/0	14														
Mnejja et al. [37]	2010	Tunisia	1990–2007	CS	R	164	LNOS		36/128	NR	32				9		8								
Csanady et al. [38]	2011	Hungary	1987–2006	CS	R	55	S		55/0	55/0	15														
Deganello et al. [10]	2011	Italy	2000–2004	CS	R	96	LNOS		55/41	82/14	12	1	10	3	2	0									
						57	S		35/22	NR	9									4	24	3	10	2	12
Deganello et al. [10]	2011	Italy	2000–2004	CS	R	39	G		20/19	NR	3											1	17	2	2
Chone et al. [39]	2012	Brazil	2007–2011	CS	P	20	LNOS		NR	NR	NR				0										
Xu et al. [40]	2012	China	1996–2009	CS	R	126	LNOS		15/111	NR	41														
Erdag et al. [41]	2013	Turkey	1996–2009	CS	R	24	G		24/0	24/0	0														
Jia et al. [42]	2013	China	2002–2010	CS	P	68	S		36/32	52/16	21						0			9	36	10	27	2	5
Furtado de Araújo Neto et al. [43]	2014	Brazil	2007–2012	CS	R	77	LNOS		0/77	NR	12				3										
Ma et al. [44]	2014	China	2002–2013	CS	R	121	S		39/82	66/55	34		22	21	4		2			6	39	13	40	15	42
Djordjevic et al. [45]	2016	Serbia	1996–2005	CC	R	193	S		107/86	NR	35		31	8	1										
Tsushima et al. [46]	2019	Japan	1998–2014	CS	R	39	LNOS		0/39	0/39	14		4	3	0		0								

CS: case series; CC: case control; G: glottis; LNOS: larynx, not otherwise specified; NR: not reported; P: prospective; R: retrospective; S: supraglottis.

## Supplementary Materials

The following are available online at https://www.mdpi.com/2072-6694/12/4/1059/s1, Figure S1: Pooled incidence of occult lymph metastasis in larynx cancer by subsite, Figure S2: Pooled incidence of occult lymph metastasis in supraglottic larynx tumor, T1–2 stage, Figure S3: Pooled incidence of occult lymph metastasis in larynx cancer, subsite NOS (not otherwise specified), T3–4 stages, Figure S4: Pooled incidence of occult lymph metastasis by levels for larynx tumor, subsite NOS (not otherwise specified), Figure S5: Pooled incidence of occult lymph metastasis by levels for supraglottic tumor, Figure S6: Pooled incidence of occult lymph metastasis for glottic tumor by stage, Figure S7: Pooled incidence of occult lymph metastasis for supraglottic tumor by stage, Table S1: Data on involvement of lymph node levels by occult neck metastasis in cN0 larynx cancer.
