# Supplementary material for: Incidence of Occult Lymph Node Metastasis in Primary Larynx Squamous Cell Carcinoma, by Subsite, T Classification and Neck Level: A Systematic Review
Source: Cancers (Basel). 2020 Apr 24;12(4):1059. doi: 10.3390/cancers12041059 (PMC7225965; doi:10.3390/cancers12041059)
Supplement: Supplementary file 1 [file cancers-12-01059-s001.pdf]

Incidence of Occult Lymph Node Metastasis in Primary Larynx Squamous Cell Carcinoma, by Subsite, T Classification and Neck Level: A Systematic Review

Alvaro Sanabria, Jatin P. Shah, Jesus E. Medina, Kerry D. Olsen, K. Thomas Robbins, Carl E. Silver, Juan P. Rodrigo, Carlos Suárez, Andrés Coca-Pelaz, Ashok R. Shaha, Antti A. Mäkitie, Alessandra Rinaldo, Remco de Bree, Primož Strojan, Marc Hamoir, Robert P. Takes, Elisabeth V. Sjögren, Trinitia Cannon, Luiz P. Kowalski and Alfio Ferlito

Table S1. Data on involvement of lymph node levels by occult neck metastasis in cN0 larynx cancer.

Level	Number of studies that evaluated the outcome	Subsite	Pooled incidence (95% CI, I2)
I	2 [10,13]	LNOS	2.4% (95% CI 0–6.1%, I2 = 54%)
II	7 [10,13,24,27,29,34,46]	LNOS	12.8% (95% CI 9.8–15.8, I2 = 27%)
II	4 [32,33,44,45]	S	25.8% (95% CI 20.1–31.6, I2 = 0)
IIb	6 [25,27,37,42,44,46]	All	0.5% (95% CI 0–1.3, I2 = 41%)
III	7 [10,13,24,27,29,34,46]	LNOS	8.3% (95% CI 4.8–11.7, I2 = 64%).
III	4 [32,33,44,45]	S	10.2% (95% CI 3.7–16.7, I2 = 76%)
IV	13 [10,13,20,24–27,29,34,37,39,43,46]	LNOS	2.0% (95% CI 0.9–3.1, I2 = 55%)
IV	4 [32,33,44,45]	S	0.9% (95% CI 0–1.7, I2 = 46%)
V	5 [10,13,20,24,34]	LNOS	0.4% (95% CI 0–1.0%, I2 = 64%)

S: supraglottic, LNOS: larynx, not otherwise specified.

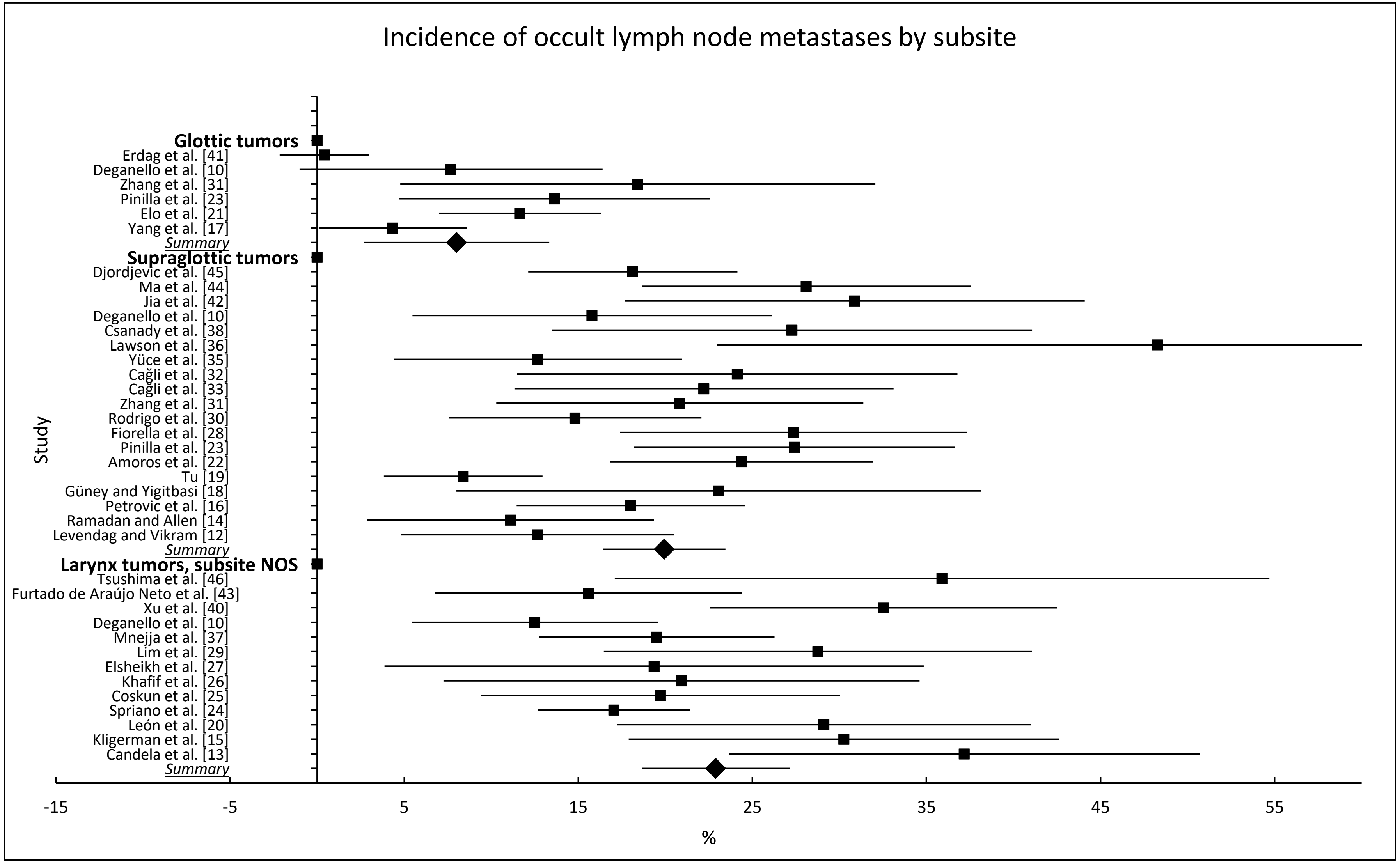

Figure S1. Pooled incidence of occult lymph metastasis in larynx cancer by subsite. NOS: not otherwise specified.

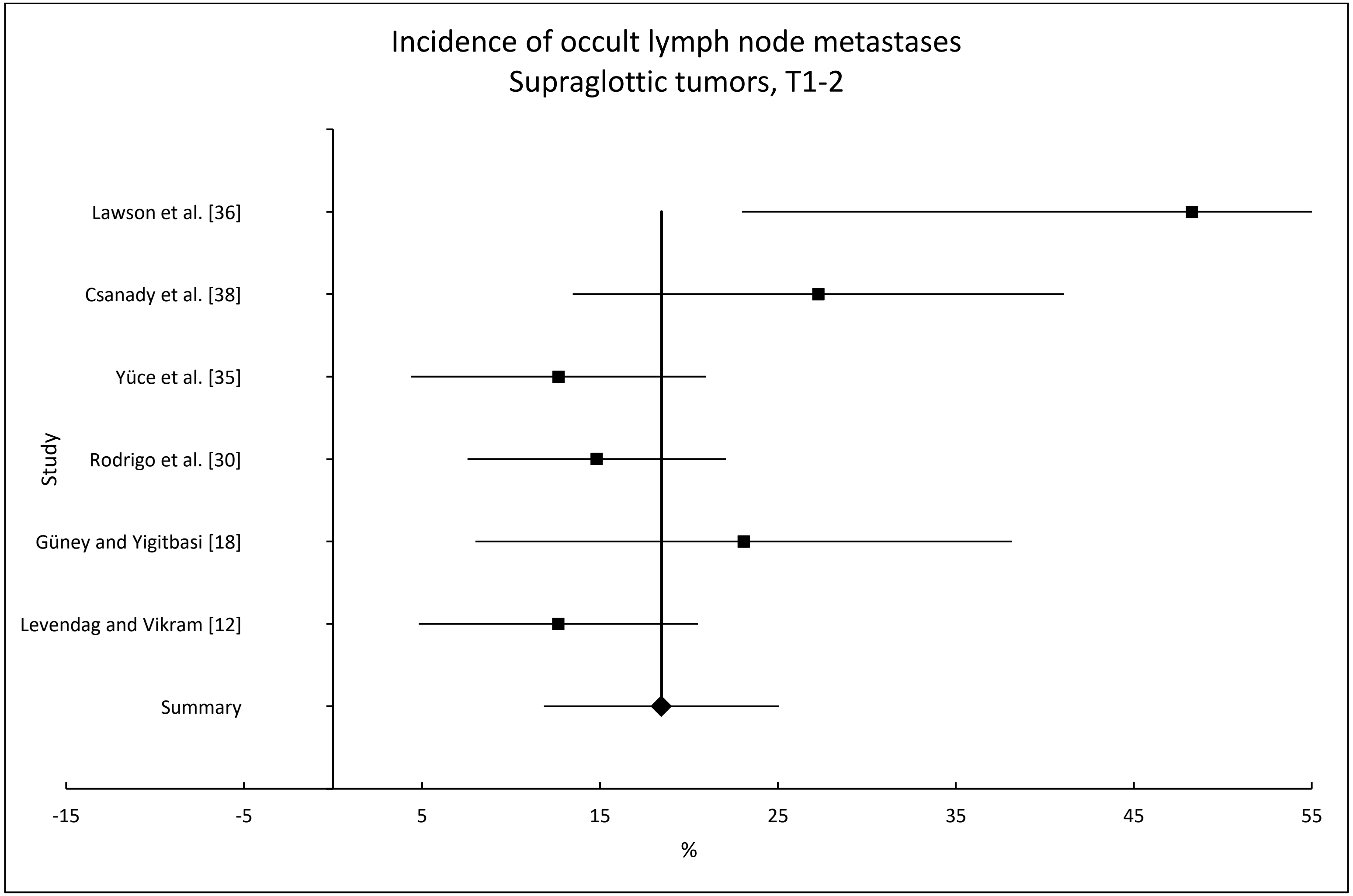

Figure S2. Pooled incidence of occult lymph metastasis in supraglottic larynx tumor, T1–2 stage.

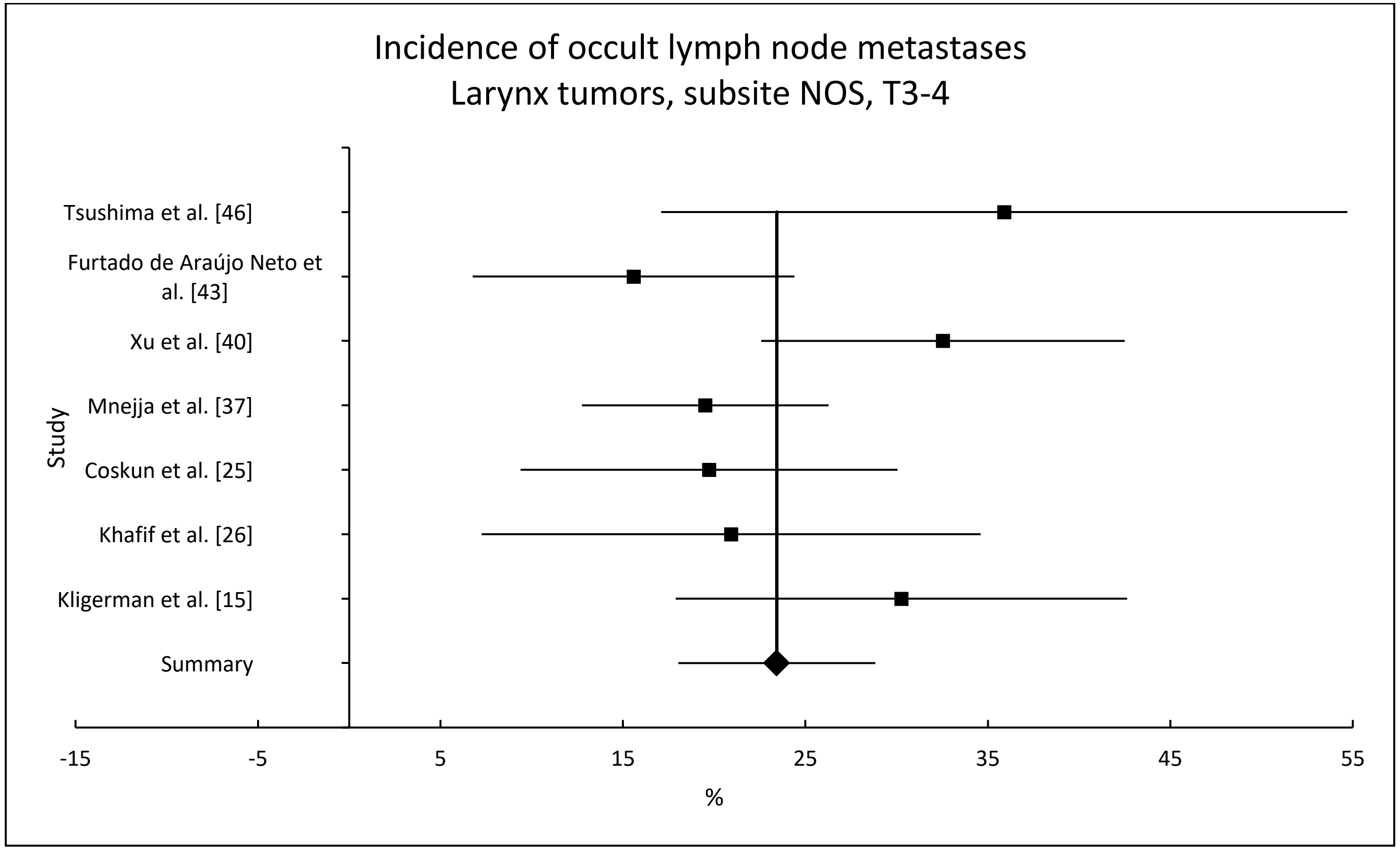

Figure S3. Pooled incidence of occult lymph metastasis in larynx cancer, subsite NOS (not otherwise specified), T3–4 stages.

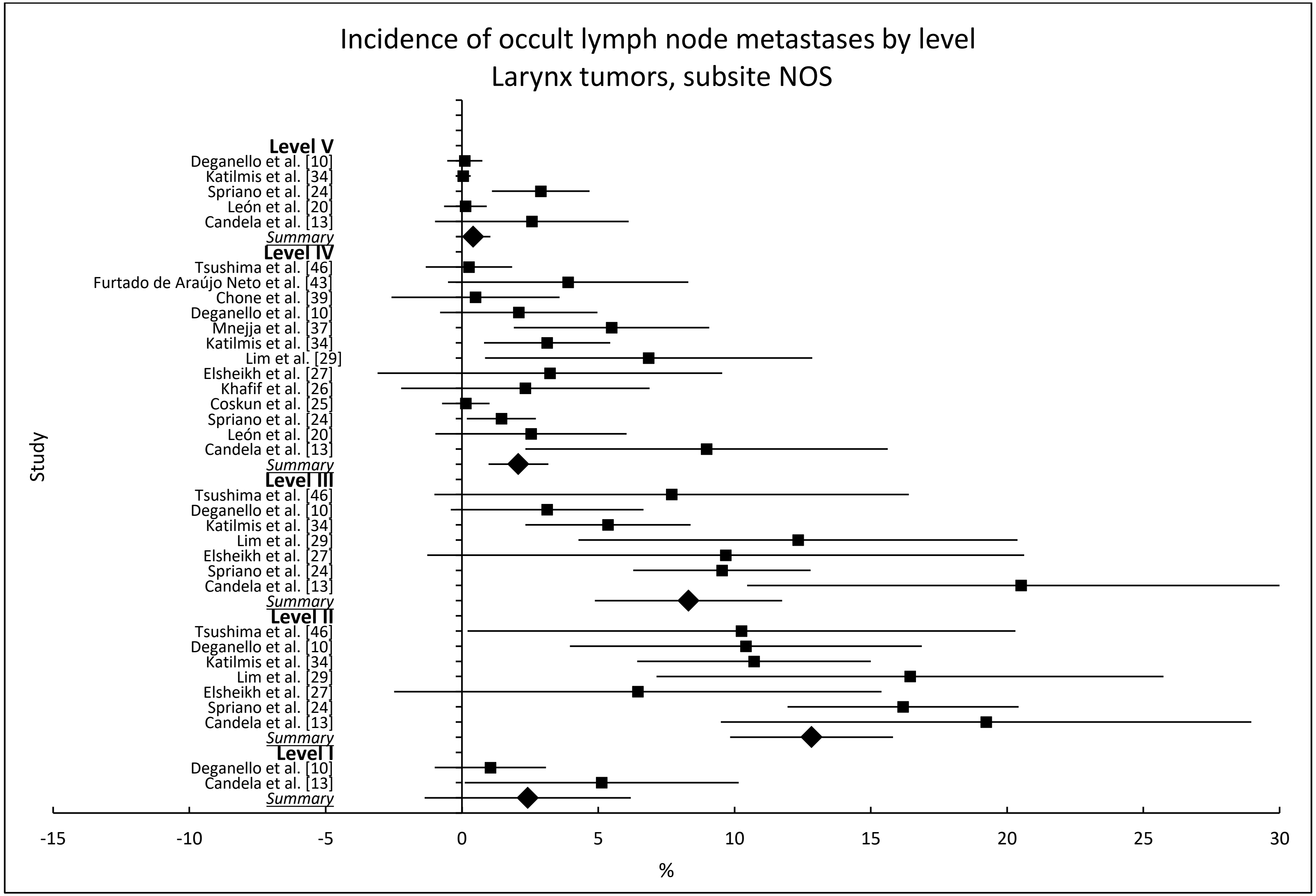

Figure S4. Pooled incidence of occult lymph metastasis by levels for larynx tumor, subsite NOS (not otherwise specified).

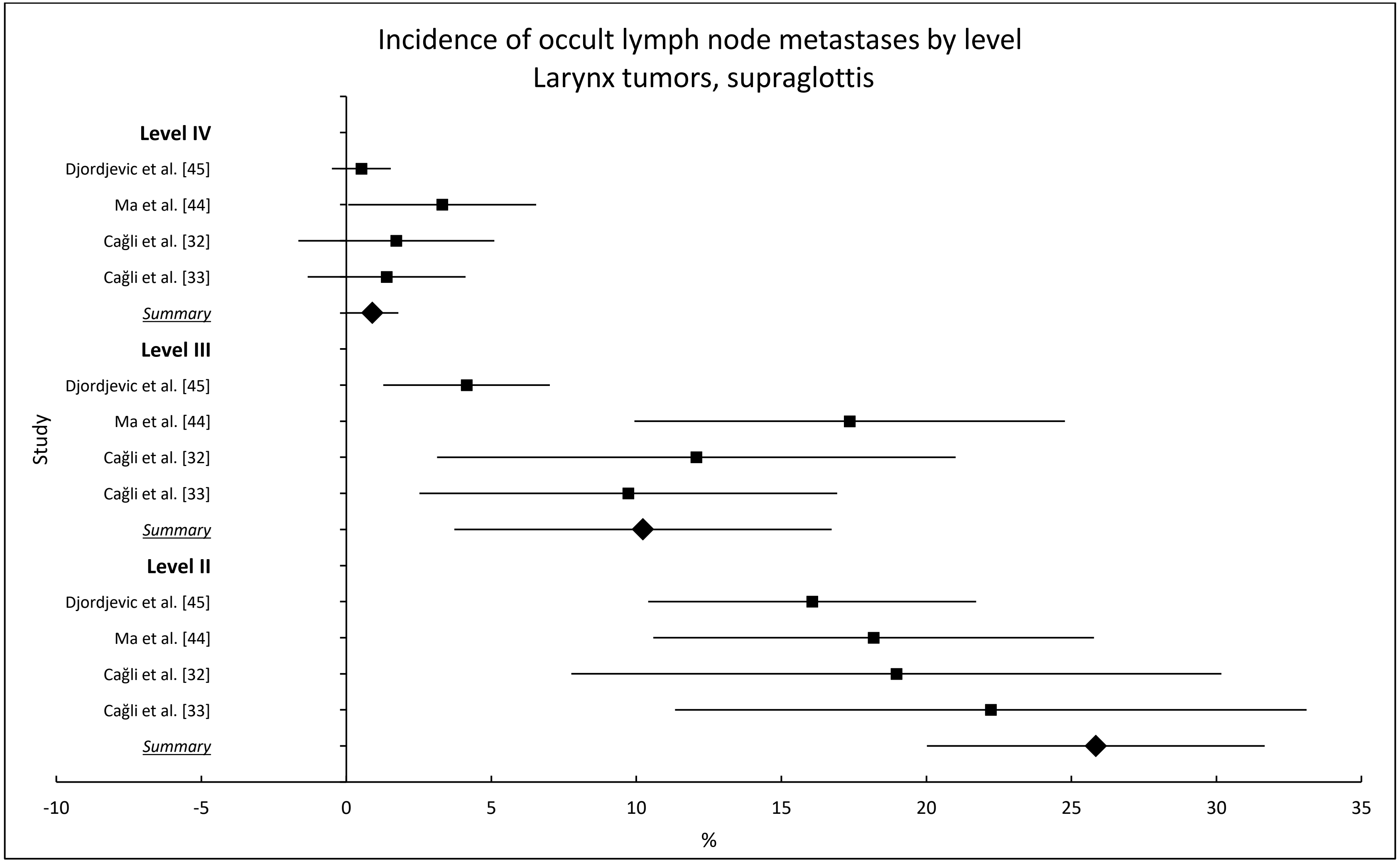

Figure S5. Pooled incidence of occult lymph metastasis by levels for supraglottic tumor.

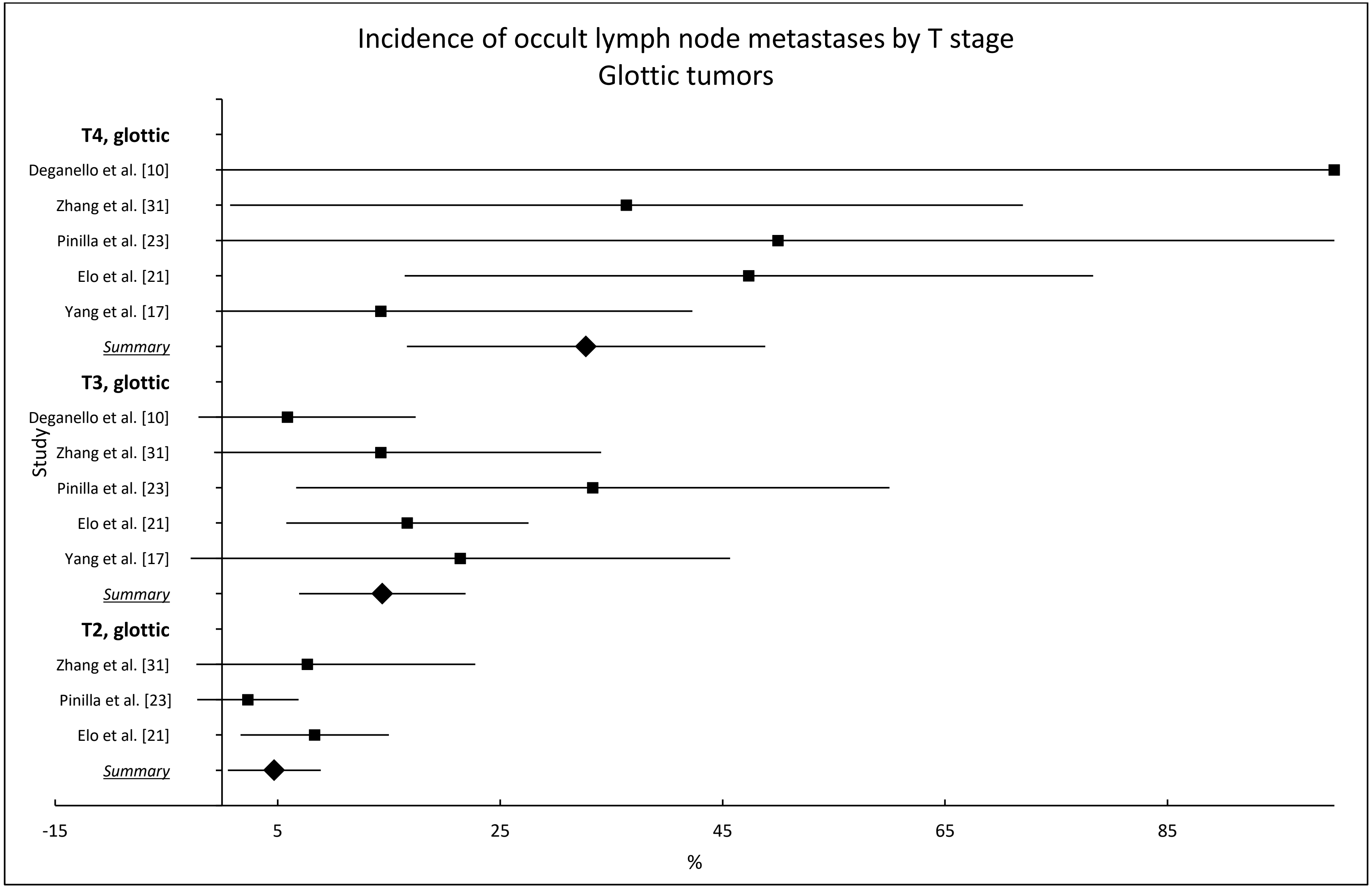

Figure S6. Pooled incidence of occult lymph metastasis for glottic tumor by stage.

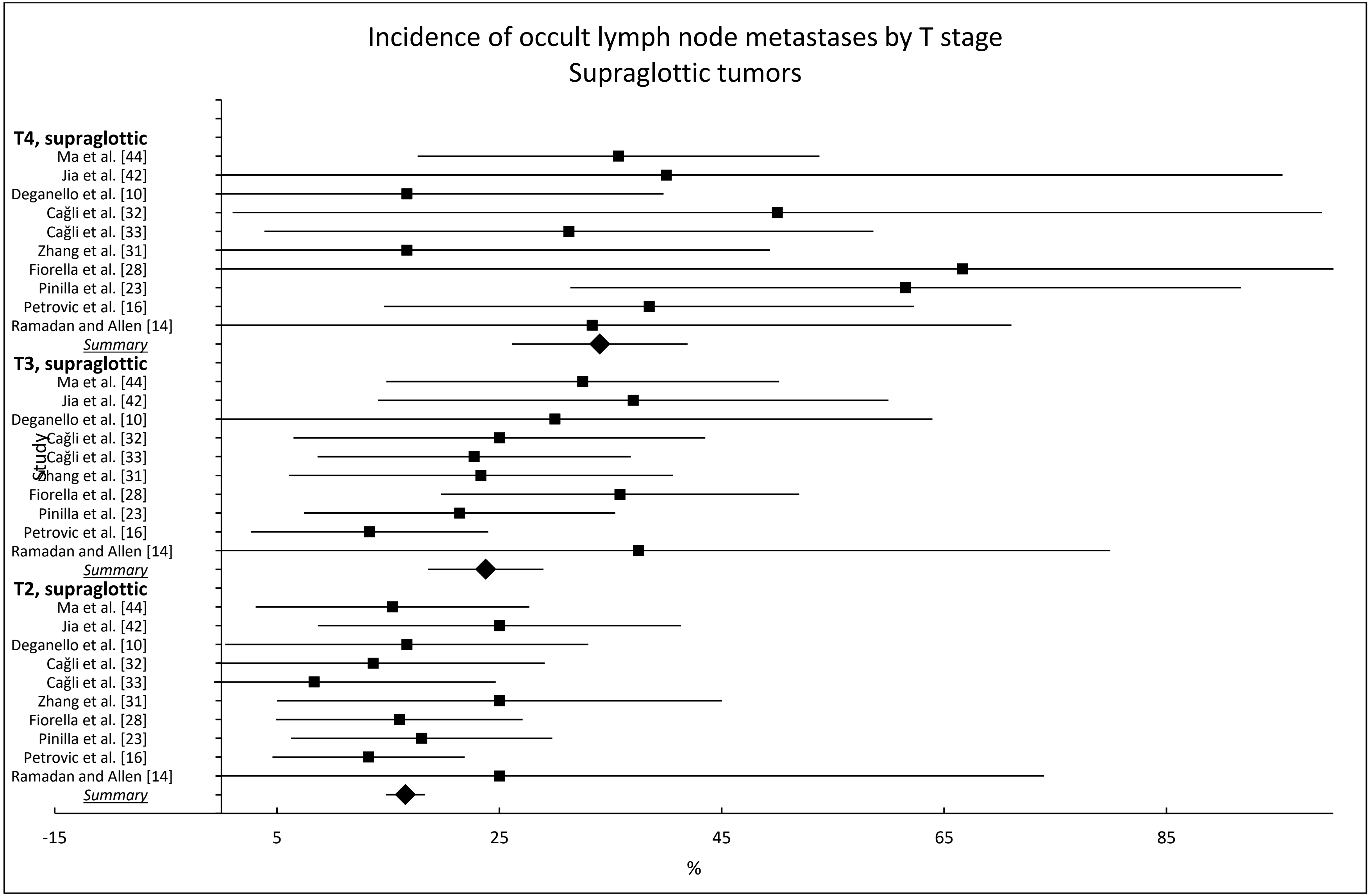
Incidence of occult lymph node metastases by T stage
Supraglottic tumors
Study
T4, supraglottic
Ma et al. [44]
Jia et al. [42]
Deganello et al. [10]
Cağli et al. [32]
Cağli et al. [33]
Zhang et al. [31]
Fiorella et al. [28]
Pinilla et al. [23]
Petrovic et al. [16]
Ramadan and Allen [14]
Summary
T3, supraglottic
Ma et al. [44]
Jia et al. [42]
Deganello et al. [10]
Cağli et al. [32]
Cağli et al. [33]
Zhang et al. [31]
Fiorella et al. [28]
Pinilla et al. [23]
Petrovic et al. [16]
Ramadan and Allen [14]
Summary
T2, supraglottic
Ma et al. [44]
Jia et al. [42]
Deganello et al. [10]
Cağli et al. [32]
Cağli et al. [33]
Zhang et al. [31]
Fiorella et al. [28]
Pinilla et al. [23]
Petrovic et al. [16]
Ramadan and Allen [14]
Summary
-15
5
25
45
65
85
%

Figure S7. Pooled incidence of occult lymph metastasis for supraglottic tumor by stage.

© 2020 by the authors. Licensee MDPI, Basel, Switzerland. This article is an open access article distributed under the terms and conditions of the Creative Commons Attribution (CC BY) license (http://creativecommons.org/licenses/by/4.0/).
